# *FXN* Promoter Silencing in the Humanized Mouse Model of Friedreich Ataxia

**DOI:** 10.1371/journal.pone.0138437

**Published:** 2015-09-22

**Authors:** Yogesh K. Chutake, Whitney N. Costello, Christina C. Lam, Aniruddha C. Parikh, Tamara T. Hughes, Michael G. Michalopulos, Mark A. Pook, Sanjay I. Bidichandani

**Affiliations:** 1 Department of Pediatrics, University of Oklahoma College of Medicine, Oklahoma City, OK 73104, United States of America; 2 Department of Biochemistry & Molecular Biology, University of Oklahoma College of Medicine, Oklahoma City, OK 73104, United States of America; 3 Ataxia Research Group, Division of Biosciences, Department of Life Sciences, College of Health & Life Sciences, Brunel University London, Uxbridge, UB8 3PH, United Kingdom; National Institute for Medical Research, Medical Research Council, London, UNITED KINGDOM

## Abstract

**Background:**

Friedreich ataxia is caused by an expanded GAA triplet-repeat sequence in intron 1 of the *FXN* gene that results in epigenetic silencing of the *FXN* promoter. This silencing mechanism is seen in patient-derived lymphoblastoid cells but it remains unknown if it is a widespread phenomenon affecting multiple cell types and tissues.

**Methodology / Principal Findings:**

The humanized mouse model of Friedreich ataxia (YG8sR), which carries a single transgenic insert of the human *FXN* gene with an expanded GAA triplet-repeat in intron 1, is deficient for *FXN* transcript when compared to an isogenic transgenic mouse lacking the expanded repeat (Y47R). We found that in YG8sR the deficiency of *FXN* transcript extended both upstream and downstream of the expanded GAA triplet-repeat, suggestive of deficient transcriptional initiation. This pattern of deficiency was seen in all tissues tested, irrespective of whether they are known to be affected or spared in disease pathogenesis, in both neuronal and non-neuronal tissues, and in cultured primary fibroblasts. *FXN* promoter function was directly measured via metabolic labeling of newly synthesized transcripts in fibroblasts, which revealed that the YG8sR mouse was significantly deficient in transcriptional initiation compared to the Y47R mouse.

**Conclusions / Significance:**

Deficient transcriptional initiation accounts for *FXN* transcriptional deficiency in the humanized mouse model of Friedreich ataxia, similar to patient-derived cells, and the mechanism underlying promoter silencing in Friedreich ataxia is widespread across multiple cell types and tissues.

## Introduction

Friedreich ataxia (FRDA) is the most common inherited ataxia and it is characterized clinically by sensory ataxia, cardiomyopathy, and a predisposition to diabetes [1]. The disease is progressive, and there is currently no effective therapy to slow the deterioration. FRDA is inherited as an autosomal recessive condition, and the vast majority of patients are homozygous for an abnormally expanded GAA triplet-repeat (GAA-TR) sequence in intron 1 of the *FXN* gene [2]. Non-FRDA alleles contain <30 triplets, while disease-causing expanded alleles typically contain 100–1300 triplets. Cells and tissues from patients who are homozygous for the expanded GAA-TR sequence have a severe deficiency of *FXN* transcript [3]. This produces a deficiency of frataxin, a mitochondrial protein that plays an important role in Fe-S cluster biogenesis [4,5], ultimately leading to pathological changes in susceptible tissues such as dorsal root ganglia, myocardium, and the cerebellar dentate nucleus [6]. A precise delineation of the mechanism(s) by which the expanded GAA-TR sequence results in transcriptional deficiency will be crucial for the development of rationally designed therapies for FRDA.

The expanded GAA-TR sequence is thought to lead to deficiency of *FXN* transcript by more than one molecular mechanism. Abnormal secondary DNA structures and repeat-proximal heterochromatin, both mediated by the expanded GAA-TR sequence, result in impedance of transcriptional elongation through intron 1 of the *FXN* gene [3,7–9]. However, the predominant mechanism of transcriptional deficiency in FRDA seems to be via epigenetic silencing of the *FXN* gene promoter [9,10]. This mechanism of silencing is reliant on the spread of repressive chromatin from the expanded GAA-TR sequence in intron 1 [9,10,11–13], which is a known source of heterochromatin [14]. This spread encompasses the *FXN* promoter, renders it transcriptionally non-permissive, and thereby causes a severe deficiency of transcriptional initiation [10]. Indeed, the length of the expanded GAA-TR sequence correlates well with the severity of *FXN* promoter silencing [15], further substantiating the etiological relationship between epigenetic silencing of the *FXN* promoter and the expanded GAA-TR mutation in intron 1. This mechanism of gene silencing, while compelling, has so far only been demonstrated in patient-derived lymphoblastoid cells [10, 15]. It remains unknown if repeat-mediated epigenetic promoter silencing is an important underlying mechanism for *FXN* transcriptional deficiency in multiple cell types and tissues, and thus its pathophysiological significance in FRDA remains unclear.

The YG8sR humanized mouse model of FRDA, which contains the entire human *FXN* gene with an expanded GAA-TR mutation in a murine *Fxn* ^-/-^ background, is known to mimic the transcriptional deficiency seen in FRDA patients [16]. Admittedly, the YG8sR mouse has a mild, variable and very late-onset phenotype. However, the deficiency of *FXN* transcript is clearly seen across multiple tissues, making it an adequate model to study the mechanism of *FXN* transcriptional deficiency in the context of the human *FXN* gene. Indeed, the mild and late-onset phenotype in the YG8sR mouse has the advantage that tissues isolated from young YG8sR mice would allow analysis prior to the onset of disease-associated pathology and thus “affected” cell types would likely be well represented in the tissue samples. The Y47R mouse [16], which has the same genomic makeup as YG8sR except that it lacks the expanded GAA-TR mutation and does not exhibit any FRDA-related phenotype, serves as a useful “non-FRDA” control.

We provide evidence that deficient transcriptional initiation accounts for the transcriptional deficiency in the YG8sR mouse model of FRDA, similar to patient-derived cells. Thus, the mechanism underlying promoter silencing in Friedreich ataxia, originally discovered in patient-derived lymphoblastoid cells, is likely widespread across multiple cell types and tissues.

## Materials and Methods

### Mouse tissues and cell lines

YG8sR and Y47R mice were bred, sacrificed and autopsied at 1 and 12 months of age at Brunel University London (U.K.) under humane conditions in accordance with the U.K. Home Office “Animals (Scientific Procedures) Act 1986” and with approval from the Brunel University London Animals Welfare and Ethical Review Board. Frozen tissue samples were sent on dry ice by overnight courier service to the University of Oklahoma Health Sciences Center for further analysis. DNA and RNA from tissues and cell lines were extracted using the DNeasy Blood and Tissue kit (Qiagen) and the RNeasy kit (Qiagen), respectively. The length of the GAA-TR sequence was analyzed by PCR amplification using previously described primers [17], and by direct sequencing of purified PCR products in both directions. Fibroblast cell lines from YG8sR and Y47R mice, established and characterized as described previously [16], were maintained in culture in DMEM supplemented with 10% FBS and penicillin and streptomycin.

### Quantitative measurement of *FXN* transcript

This was done by reverse transcription followed by quantitative polymerase chain reaction (quantitative RT-PCR), using protocols and primer sequences as previously described [10]. Briefly, reverse transcription was performed either with a mixture of random hexamers and oligo dT primers (for the Exon 3–4 amplicon; “Ex3-Ex4”) or with a strand-specific RT primer (for the Exon 1 amplicon; “Ex1”) using the QuantiTect^®^ reverse transcription kit (Qiagen). Transcript levels were quantified by real-time PCR and normalized relative to expression of the control *Tbp* gene (forward primer: 5’-CCTTGTACCCTTCACCAATGAC-3’, reverse primer: 5’-ACAGCCAAGATTCACGGTAGA-3’) for experiments using fibroblasts, and normalized relative to the geometric mean of the control genes *Psmd4* (forward primer: 5’-TGGAGAGCACTATGGTTTGTGT-3’; reverse primer: 5’-ACGTTATTCTCAGGGTTGCTTC-3’) and *Eef2* (forward primer: 5’-TGTCAGTCATCGCCCATGTG-3’; reverse primer: 5’-CATCCTTGCGAGTGTCAGTGA-3’) [18] for mouse tissues, using the ΔΔCt method on the LightCycler^®^ 96 Real-Time PCR system (Roche Diagnostics Corp) with SsoAdvanced™ Universal SYBR^®^ Green Supermix (BioRad) or *Power* SYBR^®^ Green PCR Master Mix (Life Technologies).

### Methylation sensitive—high resolution melting (MS-HRM) assay to test for CpG DNA methylation

**Fig 1:**
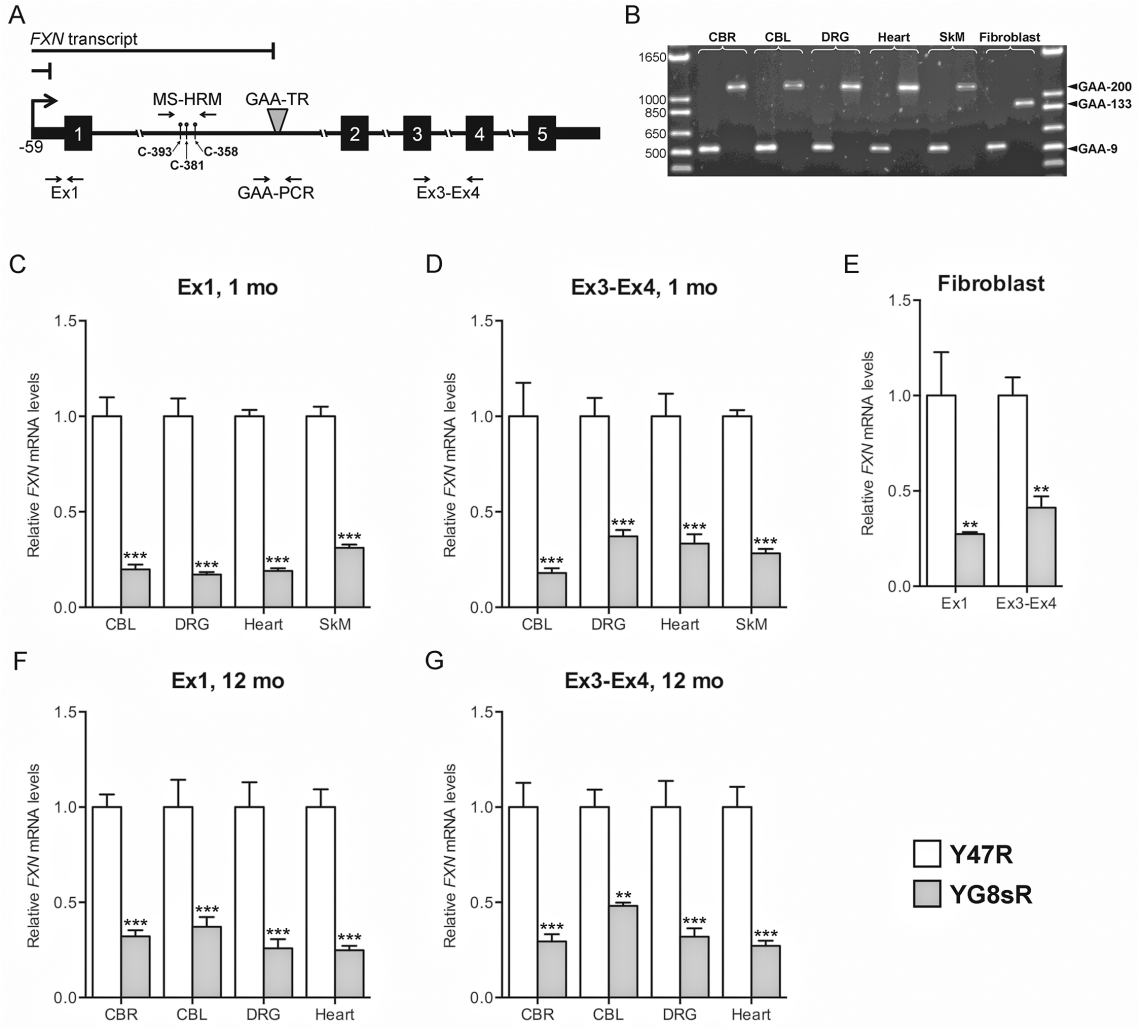
*FXN* transcriptional deficiency in the YG8sR mouse extends both upstream and downstream of the expanded GAA-TR mutation. **(A)** Relevant portions of the *FXN* gene are depicted schematically, with the GAA-TR mutation in intron 1, the *FXN* transcriptional start site (arrow) at position -59 relative to the initiation codon (“A” in ATG as +1), the three CpG sites in intron 1 used for DNA methylation analysis (relative to the first “G” in the GAA-TR sequence). Quantitative RT-PCR was performed to measure *FXN* transcript both upstream (Ex1; immediately downstream of the transcriptional start site) and downstream (Ex3-Ex4) of the GAA-TR mutation. Amplicons used for measuring the length of the GAA-TR sequence (GAA-PCR) and for methylation sensitive—high resolution melting (MS-HRM) are also depicted. Solid lines above the gene depict the shorter predicted *FXN* transcripts caused by defects in transcriptional elongation through the expanded GAA-TR mutation and by deficient transcriptional initiation due to *FXN* promoter silencing. Deficiency of transcript at both upstream and downstream locations would suggest a defect in transcriptional initiation, and deficiency of only Ex3-Ex4 would suggest a defect in transcriptional elongation. **(B)** PCR analysis to measure the length of the GAA-TR sequence in intron 1 of the *FXN* gene in various tissues from Y47R and YG8sR mice (for each tissue, the paired samples depict Y47R and YG8sR in the left and right lanes, respectively). The precise length of the GAA-9 product from Y47R fibroblasts and the GAA-133 product from YG8sR fibroblasts were determined by direct sequencing, which also showed that the repeat tract was pure (i.e., absence of non-GAA repeat sequence). **(C, D)** Quantitative RT-PCR showing deficiency of *FXN* transcript in 1-month-old YG8sR mouse tissues compared to Y47R, both upstream (Ex1) and downstream (Ex3-Ex4) of the expanded GAA-TR sequence. **(E)** Quantitative RT-PCR showing deficiency of *FXN* transcript in fibroblasts from YG8sR compared to Y47R, both upstream (Ex1) and downstream (Ex3-Ex4) of the expanded GAA-TR sequence. **(F, G)** Quantitative RT-PCR showing deficiency of *FXN* transcript in 12-month-old YG8sR mouse tissues compared to Y47R, both upstream (Ex1) and downstream (Ex3-Ex4) of the expanded GAA-TR sequence. CBR = cerebrum; CBL = cerebellum; DRG = dorsal root ganglia; SkM = skeletal muscle. Data shown in panels C through G represent three complete experiments using tissues isolated from two YG8sR and two Y47R individuals. Error bars represent +/-SEM. ** = *p*<0.01, *** = *p*<0.001.

The MS-HRM assay [19] is based on differential melting of PCR products containing different amounts of C’s and T’s following bisulfite treatment of DNA that contains differentially methylated CpG sites. DNA is amplified in the presence of a DNA binding saturating fluorescent dye, e.g. ResoLight. As the temperature of the PCR products is raised the DNA begins to melt. In bisulfite treated DNA with high endogenous CpG methylation, the C’s that remain unconverted requires a longer time & higher temperature to melt compared with those with lower CpG methylation. Primers for the MS-HRM assay were designed to amplify the bisulfite converted DNA template. Accordingly, primers were designed to amplify a 90 bp region of interest that includes CpG-393, CpG-381 and CpG-358 (forward primer: 5’-CCAAAAAAATAATAAAAAATATTTC-3’; reverse primer: 5’-ATTAGTTATTTTGAAGGGATTTTTTT-3’) [see Fig 1A]. Since depurination during bisulfite treatment damages the DNA template, longer than usual annealing and extension times (2 minutes each), and 40 cycles of amplification were performed. Genomic DNA from lymphoblastoid cell lines from three non-FRDA (GM22647, GM22671, GM15851 [Coriell Cell Repositories]) and three FRDA (GM16798, GM16197, GM14518 [Coriell Cell Repositories]) individuals homozygous for repeats containing >400 repeats, and genomic DNA from mouse-derived fibroblasts and multiple tissues from 1-month-old and 12-month old Y47R and YG8sR mice were analyzed. Purified DNA was sonicated using the EpiShear™ sonicator (Active Motif) to an average size of ~1000 bp and treated with bisulfite at 50°C for at least 5 hours using the Bisulfite Converstion Kit (Active Motif) according to the manufacturer’s protocol. MS-HRM PCR was performed using 10 ng of bisulfite treated DNA with AmpliTaq Gold^®^ DNA polymerase (Life Technologies) supplemented with 1x LightCycler^®^ 480 ResoLight dye (Roche Diagnostics Corp). Reference templates, to simulate 100% and 0% CpG methylation at the three sites, were generated by PCR using the same primers but starting with single-stranded long synthetic oligonucleotides either containing all three CpG sites intact (100% methylated) or all three CpG sites converted to TpG (0% methylated). Differential DNA melting was monitored by generating normalized melting curves on the LightCycler^®^ 96 Real-Time PCR system (Roche Diagnostics Corp) by measuring fluorescence 15 times per°C change between 65°C and 97°C, using algorithms supplied by the manufacturer.

### Quantitative measurement of *FXN* promoter activity

This was done by metabolic labeling of nascent RNA using the Click-iT^®^ Nascent RNA Capture Kit (Life Technologies) as previously described [10]. Briefly, fibroblast cell lines were incubated with 5-ethynyl uridine. Following 1, 2 and 4 hours of incubation, RNA was extracted and used for biotinylation by Click reaction. Biotinylated RNA bound to streptavidin beads was reverse transcribed using the SuperScript^®^ VILO™ cDNA synthesis kit (Life Technologies). Nascent transcript levels were quantified by real-time PCR relative to expression of the control *Tbp* gene from total biotinylated RNA using the ΔΔCt method on the LightCycler^®^ 96 Real-Time PCR system (Roche Diagnostics Corp) with SsoAdvanced™ Universal SYBR^®^ Green Supermix (BioRad) or *Power* SYBR^®^ Green PCR Master Mix (Life Technologies).

## Results

### *FXN* transcriptional deficiency in the YG8sR mouse extends upstream and downstream of the expanded GAA-TR mutation

Multiple tissues (cerebrum, cerebellum, dorsal root ganglia [DRG], heart, and skeletal muscle) and fibroblast cell lines derived from YG8sR and Y47R mice were selected for analysis of *FXN* transcript levels both upstream and downstream of the expanded GAA-TR mutation in order to differentiate between defects in transcriptional initiation and transcriptional elongation through the expanded repeat. Deficiency of transcript at both locations would suggest a defect in transcriptional initiation, but deficiency at only the downstream location would suggest a defect in transcriptional elongation (Fig 1A). The tissues and cell lines were selected to represent various characteristics, including those that are known to be affected versus unaffected in FRDA, neuronal versus non-neuronal and proliferative versus post-mitotic. All tissues and cell lines from the transgenic mouse lines contained a single GAA-TR length; Y47R tissues and fibroblasts contained 9 triplets, YG8sR tissues contained ~200 triplets, and YG8sR fibroblasts contained 133 GAA triplets (the latter was confirmed by sequencing to be a pure GAA triplet-repeat [16]; Fig 1B).

For the upstream location, quantitative RT-PCR was performed in the immediate vicinity of the transcription start site of the *FXN* gene (“Ex1”), and the spliced product of exons 3 & 4 (“Ex3-Ex4”) was selected as the downstream location (Fig 1A). We found significant deficiency of *FXN* transcript in YG8sR compared with Y47R at both the upstream and downstream locations in all tissues and cell lines tested (Fig 1C–1G), supporting the existence of a defect in transcriptional initiation, as was previously noted in FRDA patient-derived lymphoblastoid cell lines [10,15]. We initially focused our analysis on 1-month-old mouse tissues (as shown in Fig 1C and 1D) in order to preempt any phenotypic manifestations in the YG8sR mouse and thus potentially representing all cell types including those that would be lost due to late-onset FRDA-associated pathology. However, we subsequently observed a similar deficiency in steady-state *FXN* transcript levels in 12-month-old YG8sR mouse tissues (Fig 1F and 1G) as we had seen in 1-month-old mouse tissues, suggesting that the mechanism of transcriptional deficiency persists throughout life. Overall, these data suggest that transcriptional deficiency in the YG8sR mouse model likely stems from deficient transcriptional initiation.

### Increased DNA methylation at the *FXN* locus in the YG8sR mouse

Among the FRDA-specific epigenetic changes at the *FXN* locus that are associated with the expanded GAA-TR mutation is increased DNA methylation at CpG sites in the vicinity of the repeat in intron 1. The level of DNA methylation at CpG-393, CpG-381 and CpG-358 (numbering refers to the nucleotide position of the “C” with respect to the first “G” in the GAA-TR sequence; see Fig 1A) is known to be increased in FRDA patients who are homozygous for the expanded GAA-TR mutation [20–22]. Indeed, methylation at CpG-381 is known to correlate with repeat length and age of disease onset [21], and methylation at CpG-358 correlates with *FXN* transcript levels, age of onset and the FARS clinical rating scale [22]. Given the correlation of CpG methylation at these sites with phenotypically-relevant features in FRDA, we investigated if YG8sR tissues and fibroblasts also showed increased CpG methylation at these sites, indicative of relevant expression-related epigenetic changes at the *FXN* locus despite the relatively short expanded GAA-TR mutation.

**Fig 2:**
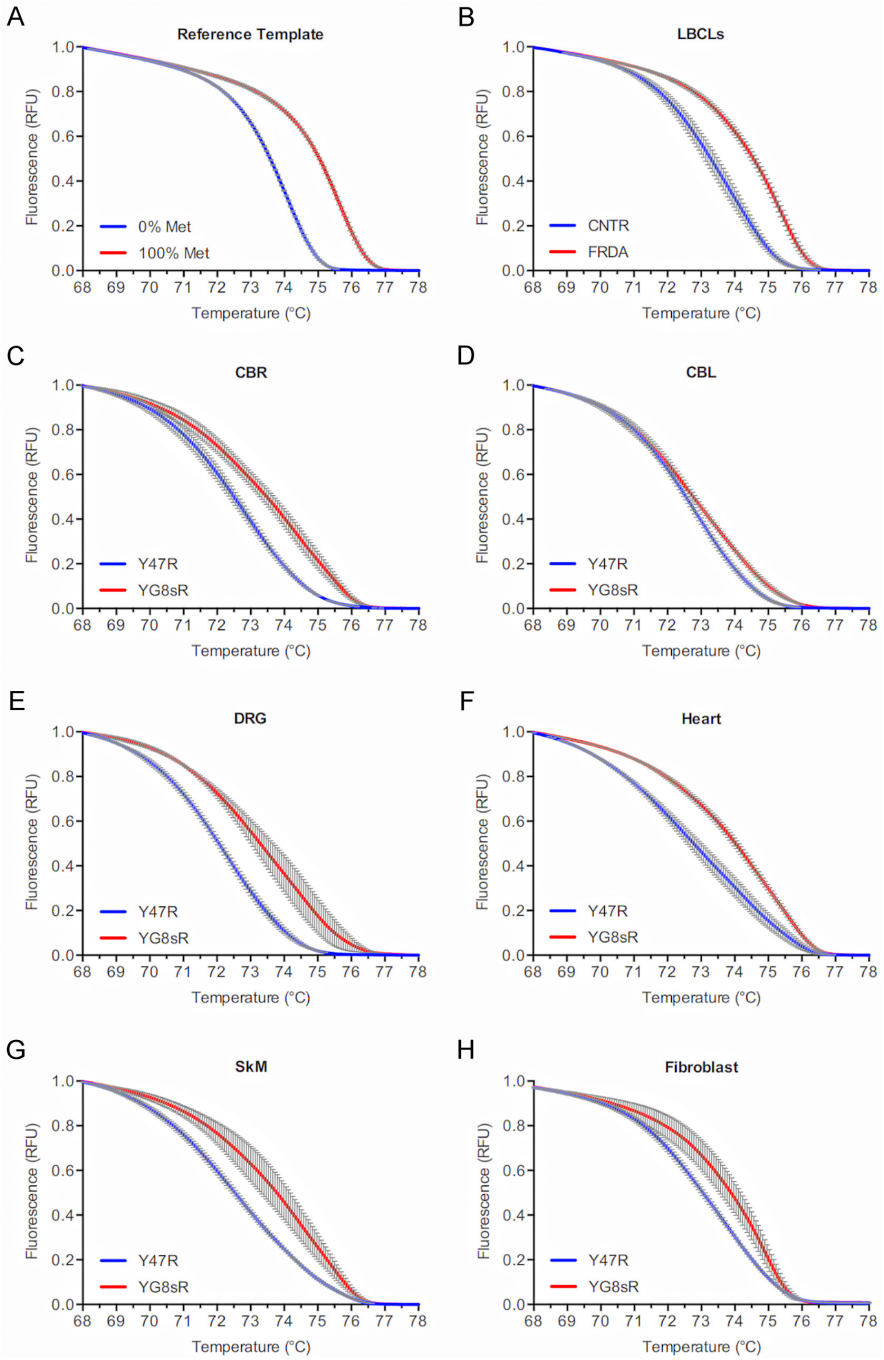
Increased DNA methylation at the *FXN* locus in the 1-month-old YG8sR mouse. **(A)** Normalized melting curves in a high resolution melting (HRM) assay of two reference double-stranded templates simulating 100% (red curve) and 0% (blue curve) DNA methylation at three CpG sites upstream of the GAA-TR mutation (see Fig 1A) showing a clear separation of the curves indicating that the HRM assay is able to detect methylation at the three CpG sites. **(B)** Normalized melting curves in a methylation sensitive—high resolution melting (MS-HRM) assay to detect CpG methylation in lymphoblastoid cell lines from three FRDA (red curve) and three non-FRDA control subjects (blue curve) at the three CpG sites upstream of the GAA-TR mutation (see Fig 1A) showing a clear separation of the curves indicating that the MS-HRM assay is able to detect a relative increase in methylation at the three CpG sites. **(C-H)** Normalized melting curves in a MS-HRM assay to detect CpG methylation in fibroblast cell lines and multiple tissues from 1-month-old YG8sR (red curves) and Y47R (blue curves) mice at the three CpG sites upstream of the GAA-TR mutation (see Fig 1A) showing a clear separation of the curves indicating a relative increase in methylation at the three CpG sites in YG8sR tissues and fibroblasts. For all HRM curves, X-axis = melting temperature, Y-axis = relative fluorescence, and error bars represent 95% confidence intervals at each of 15 points assayed in triplicate for fluorescence per°C change. LBCLs = lymphoblastoid cell lines; CBR = cerebrum; CBL = cerebellum; DRG = dorsal root ganglia; SkM = skeletal muscle.

**Fig 3:**
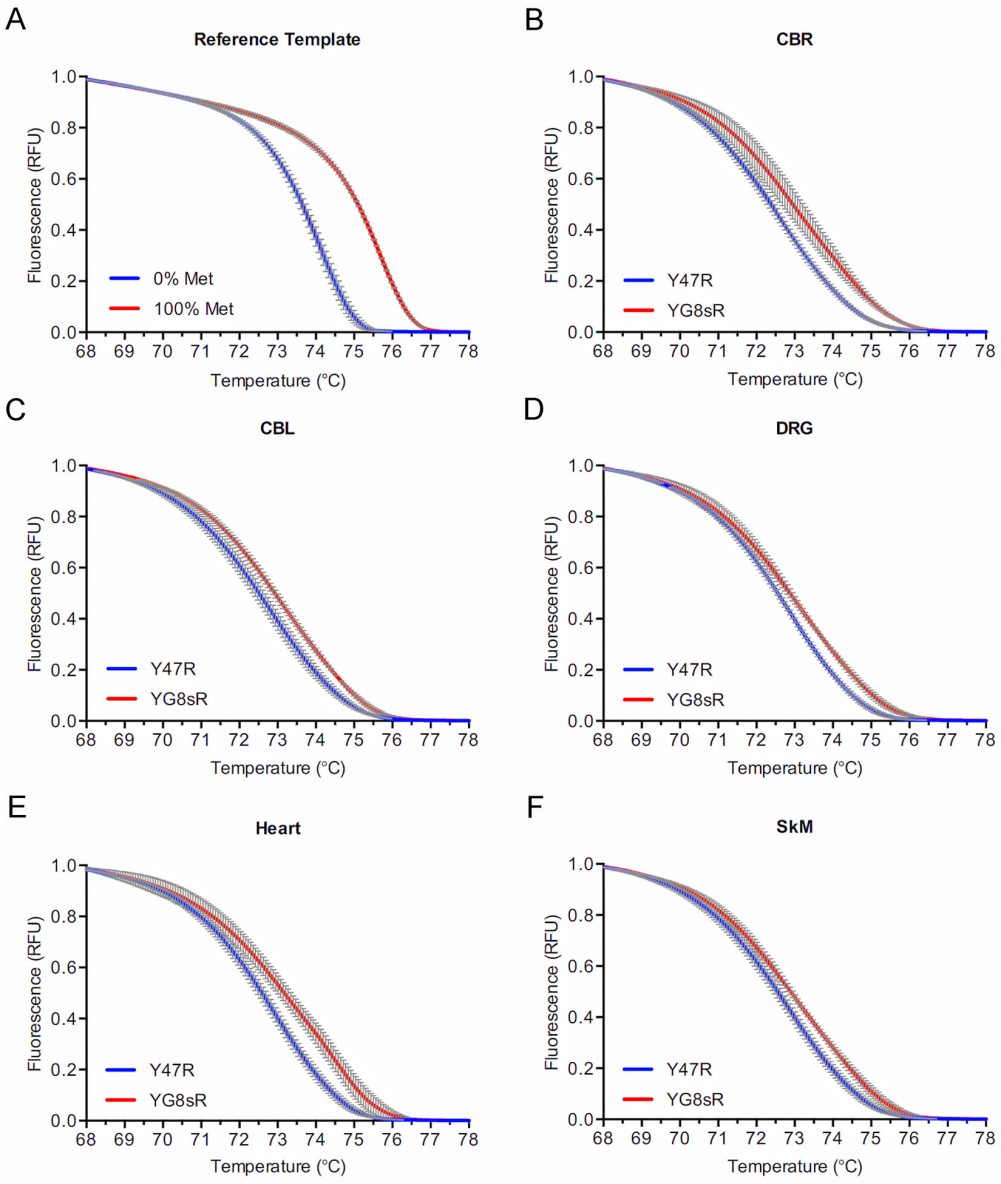
Increased DNA methylation at the *FXN* locus in the 12-month-old YG8sR mouse. **(A)** Normalized melting curves in a high resolution melting (HRM) assay of two reference double-stranded templates simulating 100% (red curve) and 0% (blue curve) DNA methylation at three CpG sites upstream of the GAA-TR mutation (see Fig 1A) showing a clear separation of the curves indicating that the HRM assay is able to detect methylation at the three CpG sites. **(B-F)** Normalized melting curves in a MS-HRM assay to detect CpG methylation in multiple tissues from 12-month-old YG8sR (red curves) and Y47R (blue curves) mice at the three CpG sites upstream of the GAA-TR mutation (see Fig 1A) showing a clear separation of the curves indicating a relative increase in methylation at the three CpG sites in YG8sR tissues. For all HRM curves, X-axis = melting temperature, Y-axis = relative fluorescence, and error bars represent 95% confidence intervals at each of 15 points assayed in triplicate for fluorescence per°C change. CBR = cerebrum; CBL = cerebellum; DRG = dorsal root ganglia; SkM = skeletal muscle.

A suitable amplicon was designed to span CpG–393, CpG-381, and CpG–358 (Fig 1A). A semi-quantitative assay involving bisulfite conversion followed by high resolution melting (methylation sensitive-high resolution melting [MS-HRM] assay [19]) was developed to assess for increased CpG methylation in YG8sR compared to Y47R (see Materials and Methods). Two positive controls were used to determine the discriminatory capacity of the MS-HRM assay. Firstly, two separate double-stranded reference templates simulating 100% methylation (i.e., C’s at the three CpG sites) and 0% methylation (i.e., T’s at the three CpG sites) were directly used for HRM analysis (i.e., without bisulfite conversion). This showed a clear separation of melting curves (Fig 2A), indicating that the HRM part of the assay was capable of detecting complete methylation at the three CpG sites. Next, we tested DNA from lymphoblastoid cell lines of FRDA patients homozygous for long GAA-TR mutations (i.e., with both expanded alleles containing >400 triplets), which are known to have increased CpG methylation compared to non-FRDA controls. This also showed a clear separation of the high resolution melting curves due to increased DNA methylation in FRDA versus non-FRDA cell lines, indicating that the MS-HRM assay was capable of detecting even relative increases in methylation at the three CpG sites contained within the amplicon (Fig 2B). The MS-HRM assay was then used to test multiple tissues from 1-month-old YG8sR and Y47R mice, and fibroblast cell lines from both mouse lines, which showed evidence of increased CpG methylation involving the three CpG sites in YG8sR (Fig 2C–2H). The MS-HRM assay also revealed a similar increase in DNA methylation in multiple tissues from two additional 1-month-old YG8sR and Y47R individuals (S1 and S2 Figs), and two 12-month-old YG8sR and Y47R individuals (Fig 3 and S3 Fig), suggesting that this epigenetic change persists throughout life. It should be noted that while this assay simultaneously measures DNA methylation at the three CpG sites in a large number of cells (as opposed to typical assays that involve sequencing of a limited number of cloned templates), it lacks the ability to detect relative methylation levels at the three individual CpG sites contained within the amplicon. These data indicate there are repressive epigenetic changes in the form of increased DNA methylation at phenotypically-relevant CpG sites located upstream of the expanded GAA-TR in intron 1 in multiple tissues and fibroblasts from YG8sR versus Y47R.

### Deficiency of *FXN* transcriptional initiation in the YG8sR mouse

**Fig 4:**
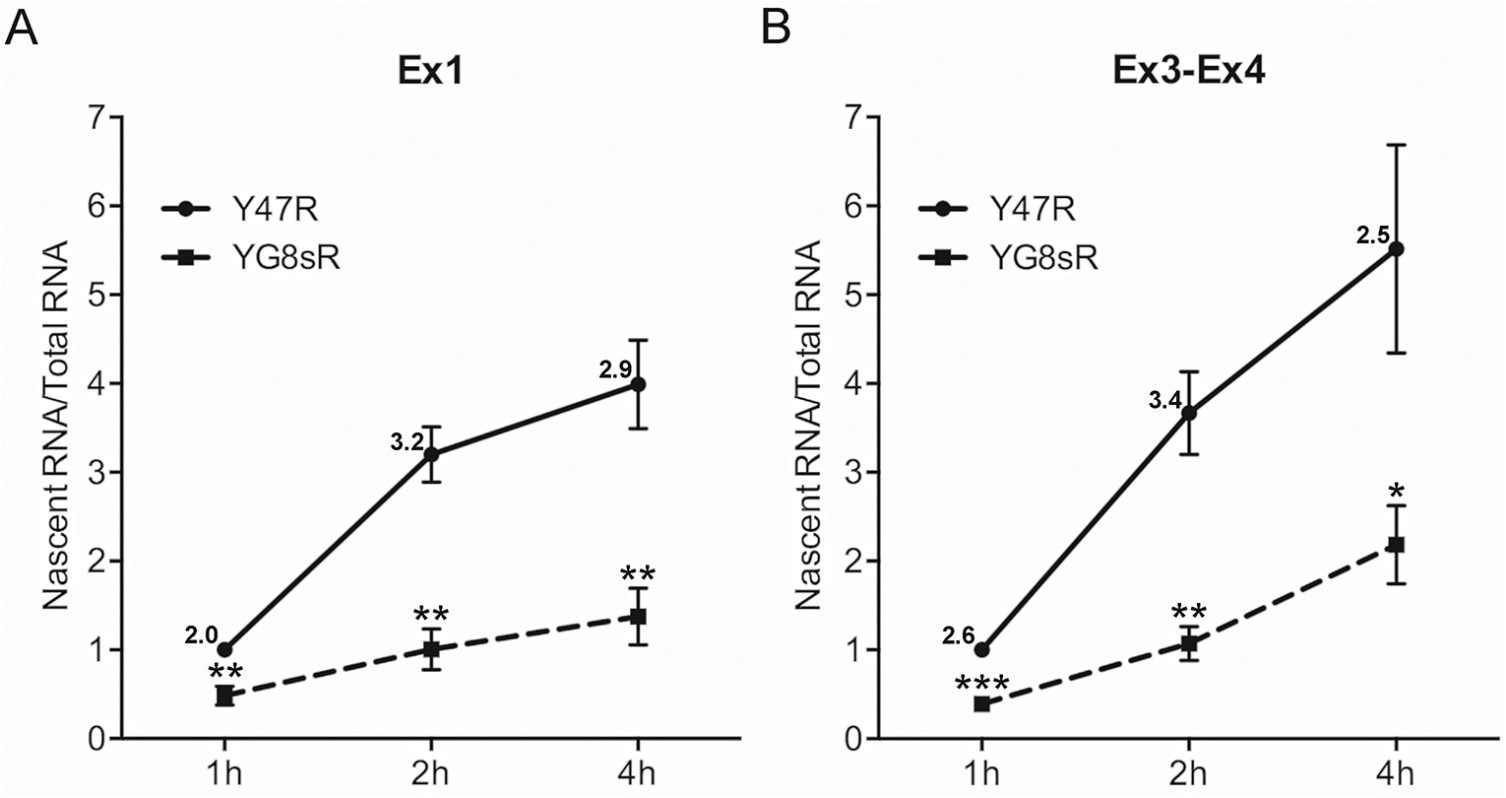
Metabolic labeling of nascent *FXN* transcript in primary fibroblasts showing deficiency of transcriptional initiation in the YG8sR mouse. **(A, B)** Quantitative RT-PCR of metabolically labeled nascent transcript for the indicated incubation times (1, 2 and 4 hours) is shown for *FXN* mRNA upstream (“Ex1” in Fig 1A) and downstream (“Ex3-Ex4” in Fig 1A) of the GAA-TR sequence in intron 1. YG8sR cells showed 2.0–3.4 fold less nascent *FXN* transcript (exact fold changes are indicated) compared with Y47R cells at all the time points assayed. Graphs represent cumulative data from four independent metabolic labeling experiments. Error bars represent +/-SEM. * = *p*<0.05; ** = *p*<0.01, *** = *p*<0.001.

The combination of repressive epigenetic changes and transcriptional deficiency extending upstream of the expanded GAA-TR mutation in tissues and fibroblasts from the YG8sR mouse model suggested that there was a deficiency of transcriptional initiation similar to the epigenetic promoter silencing in lymphoblastoid cells from FRDA patients [10,15]. To directly test if the *FXN* promoter is rendered less active in YG8sR, *FXN* transcriptional initiation was measured via metabolic labeling of nascent transcripts in fibroblast cell lines derived from YG8sR and Y47R mice. Nascent transcripts were labeled with ethynyl uridine, to which biotin was subsequently added via click chemistry, thus permitting a quantitative, dynamic, *in vivo* analysis of newly synthesized *FXN* transcript. Quantitative RT-PCR was performed to measure *FXN* transcript levels both upstream (“Ex1” which maps immediately downstream of the *FXN* transcription start site [*FXN*-TSS; Fig 1A]) and downstream (“Ex3-Ex4” [Fig 1A]) of the expanded GAA-TR mutation following 1, 2, and 4 hours of labeling. This revealed a significant, 2.0 to 3.4-fold deficiency of newly synthesized *FXN* transcript in YG8sR at both locations (Fig 4A & 4B). The deficiency of dynamic accumulation of *FXN* transcript levels observed immediately downstream of *FXN*-TSS (and upstream of the expanded GAA-TR mutation) indicates that the YG8sR mouse is deficient in transcriptional initiation. Moreover, the fold-difference in accumulation of *FXN* transcript at the various time points in YG8sR versus Y47R were similar at both the upstream and downstream locations (Fig 4A & 4B), suggesting that deficient transcriptional initiation likely accounts for most of the transcriptional deficiency in the YG8sR mouse. Thus transcriptional deficiency in the YG8sR mouse model is largely due to *FXN* promoter silencing, which leads to deficiency of transcriptional initiation, similar to lymphoblastoid cell lines from FRDA patients.

## Discussion

Our results indicate that *FXN* transcriptional deficiency in the YG8sR humanized mouse model of FRDA is caused by deficient transcriptional initiation as a result of promoter silencing. While this mechanism has previously been noted in patient-derived lymphoblastoid cell lines [10,15], our present data provide supportive evidence for the existence of this mechanism of transcriptional deficiency in fibroblasts and in multiple tissues. Our data also suggest that the mechanism underlying *FXN* transcriptional deficiency in FRDA is unlikely to be tissue-specific.

It is noteworthy that tissues from the YG8sR mouse have a GAA-TR length of ~200 triplets and the fibroblast cell line contains only 133 triplets [16]. In FRDA patients, these shorter than average repeat lengths would be expected to result in a later age of onset [23] and a slowly progressive clinical phenotype [24,25]. It is therefore not surprising that the YG8sR mouse has a phenotype that is mild, variable, and of late onset [16]. Indeed, the 2- to 3-fold reduction in promoter activity in the YG8sR mouse is comparable to the magnitude of deficiency of transcriptional initiation seen in cell lines from FRDA patients who have at least one short GAA-TR allele (containing <400 GAA triplets) [15]. Therefore, a humanized mouse model based on YG8sR but containing >400 GAA triplets would likely result in more severe promoter silencing and possibly lead to a more discernable FRDA-related phenotype.

Our data indicate that the YG8sR humanized mouse is a reasonable model for investigating the molecular mechanism(s) underlying repeat-mediated promoter silencing in FRDA. The YG8sR mouse model would also be useful for testing drugs that are designed to reverse the transcriptional initiation defect caused by promoter silencing in FRDA, such as the 2-aminobenzamide derived histone deacetylase inhibitors [11,26–28].

## Supporting Information

S1 Fig Increased DNA methylation at the *FXN* locus in 1-month-old YG8sR mouse tissues. (PDF)

S2 Fig Increased DNA methylation at the *FXN* locus in 1-month-old YG8sR mouse tissues. (PDF)

S3 Fig Increased DNA methylation at the *FXN* locus in 12-month-old YG8sR mouse tissues. (PDF)

## References

[1] Bidichandani SI, Delatycki MB. Friedreich Ataxia. In: Pagon RA, Adam MP, Ardinger HH, Wallace SE, Amemiya A, Bean LJH, et al., editors. GeneReviews(R). Seattle (WA)1993 [updated 2014]. 20301458

[2] Campuzano V, Montermini L, Molto MD, Pianese L, Cossee M, Cavalcanti F, et al Friedreich's ataxia: autosomal recessive disease caused by an intronic GAA triplet repeat expansion. Science. 1996;271(5254):1423–7. 8596916 10.1126/science.271.5254.1423

[3] Bidichandani SI, Ashizawa T, Patel PI. The GAA triplet-repeat expansion in Friedreich ataxia interferes with transcription and may be associated with an unusual DNA structure. Am J Hum Genet. 1998;62(1):111–21. 9443873 10.1086/301680 PMC1376805

[4] Colin F, Martelli A, Clemancey M, Latour JM, Gambarelli S, Zeppieri L, et al Mammalian frataxin controls sulfur production and iron entry during de novo Fe4S4 cluster assembly. J Am Chem Soc. 2013;135(2):733–40. 10.1021/ja308736e 23265191

[5] Bridwell-Rabb J, Fox NG, Tsai CL, Winn AM, Barondeau DP. Human frataxin activates Fe-S cluster biosynthesis by facilitating sulfur transfer chemistry. Biochemistry. 2014;53(30):4904–13. 10.1021/bi500532e 24971490 PMC4215901

[6] Koeppen AH, Mazurkiewicz JE. Friedreich ataxia: neuropathology revised. J Neuropathol Exp Neurol. 2013;72(2):78–90. 10.1097/NEN.0b013e31827e5762 23334592 PMC3817014

[7] Ohshima K, Montermini L, Wells RD, Pandolfo M. Inhibitory effects of expanded GAA.TTC triplet repeats from intron I of the Friedreich ataxia gene on transcription and replication in vivo. J Biol Chem. 1998;273(23):14588–95. 9603975 10.1074/jbc.273.23.14588

[8] Sakamoto N, Ohshima K, Montermini L, Pandolfo M, Wells RD. Sticky DNA, a self-associated complex formed at long GAA*TTC repeats in intron 1 of the frataxin gene, inhibits transcription. J Biol Chem. 2001;276(29):27171–7. 11340071 10.1074/jbc.M101879200

[9] Kumari D, Biacsi RE, Usdin K. Repeat expansion affects both transcription initiation and elongation in friedreich ataxia cells. J Biol Chem. 2011;286(6):4209–15. 10.1074/jbc.M110.194035 21127046 PMC3039332

[10] Chutake YK, Costello WN, Lam C, Bidichandani SI. Altered Nucleosome Positioning at the Transcription Start Site and Deficient Transcriptional Initiation in Friedreich Ataxia. J Biol Chem. 2014;289(22):15194–202. 10.1074/jbc.M114.566414 24737321 PMC4140879

[11] Herman D, Jenssen K, Burnett R, Soragni E, Perlman SL, Gottesfeld JM. Histone deacetylase inhibitors reverse gene silencing in Friedreich's ataxia. Nat Chem Biol. 2006;2(10):551–8. 16921367 10.1038/nchembio815

[12] Punga T, Buhler M. Long intronic GAA repeats causing Friedreich ataxia impede transcription elongation. EMBO molecular medicine. 2010;2(4):120–9. 10.1002/emmm.201000064 20373285 PMC3377279

[13] Kim E, Napierala M, Dent SY. Hyperexpansion of GAA repeats affects post-initiation steps of FXN transcription in Friedreich's ataxia. Nucleic Acids Res. 2011;39(19):8366–77. 10.1093/nar/gkr542 21745819 PMC3201871

[14] Saveliev A, Everett C, Sharpe T, Webster Z, Festenstein R. DNA triplet repeats mediate heterochromatin-protein-1-sensitive variegated gene silencing. Nature. 2003;422(6934):909–13. 12712207 10.1038/nature01596

[15] Chutake YK, Lam C, Costello WN, Anderson M, Bidichandani SI. Epigenetic promoter silencing in Friedreich ataxia is dependent on repeat length. Ann Neurol. 2014;76(4):522–8. 10.1002/ana.24249 25112975 PMC4191993

[16] Anjomani Virmouni S, Ezzatizadeh V, Sandi C, Sandi M, Al-Mahdawi S, Chutake Y, et al A novel GAA-repeat-expansion-based mouse model of Friedreich's ataxia. Dis Model Mech. 2015;8(3):225–35. 10.1242/dmm.018952 25681319 PMC4348561

[17] Filla A, De Michele G, Cavalcanti F, Pianese L, Monticelli A, Campanella G, et al The relationship between trinucleotide (GAA) repeat length and clinical features in Friedreich ataxia. Am J Hum Genet. 1996;59(3):554–60. 8751856 PMC1914893

[18] Kouadjo KE, Nishida Y, Cadrin-Girard JF, Yoshioka M, St-Amand J. Housekeeping and tissue-specific genes in mouse tissues. BMC Genomics. 2007;8:127 17519037 10.1186/1471-2164-8-127 PMC1888706

[19] Wojdacz TK, Dobrovic A, Hansen LL. Methylation-sensitive high-resolution melting. Nat Protoc. 2008;3(12):1903–8. 10.1038/nprot.2008.191 19180074

[20] Greene E, Mahishi L, Entezam A, Kumari D, Usdin K. Repeat-induced epigenetic changes in intron 1 of the frataxin gene and its consequences in Friedreich ataxia. Nucleic Acids Res. 2007;35(10):3383–90. 17478498 10.1093/nar/gkm271 PMC1904289

[21] Castaldo I, Pinelli M, Monticelli A, Acquaviva F, Giacchetti M, Filla A, et al DNA methylation in intron 1 of the frataxin gene is related to GAA repeat length and age of onset in Friedreich ataxia patients. J Med Genet. 2008;45(12):808–12. 10.1136/jmg.2008.058594 18697824

[22] Evans-Galea MV, Carrodus N, Rowley SM, Corben LA, Tai G, Saffery R, et al FXN methylation predicts expression and clinical outcome in Friedreich ataxia. Ann Neurol. 2012;71(4):487–97. 10.1002/ana.22671 22522441

[23] Durr A, Cossee M, Agid Y, Campuzano V, Mignard C, Penet C, et al Clinical and genetic abnormalities in patients with Friedreich's ataxia. N Engl J Med. 1996;335(16):1169–75. 8815938 10.1056/NEJM199610173351601

[24] Regner SR, Wilcox NS, Friedman LS, Seyer LA, Schadt KA, Brigatti KW, et al Friedreich ataxia clinical outcome measures: natural history evaluation in 410 participants. Journal of Child Neurology. 2012;27(9):1152–8. 10.1177/0883073812448462 22752494 PMC3674496

[25] Metz G, Coppard N, Cooper JM, Delatycki MB, Durr A, Di Prospero NA, et al Rating disease progression of Friedreich's ataxia by the International Cooperative Ataxia Rating Scale: analysis of a 603-patient database. Brain. 2013;136(Pt 1):259–68. 10.1093/brain/aws309 23365101 PMC3624678

[26] Soragni E, Miao W, Iudicello M, Jacoby D, De Mercanti S, Clerico M, et al Epigenetic therapy for Friedreich ataxia. Ann Neurol. 2014;76(4):489–508. 10.1002/ana.24260 25159818 PMC4361037

[27] Sandi C, Pinto RM, Al-Mahdawi S, Ezzatizadeh V, Barnes G, Jones S, et al Prolonged treatment with pimelic o-aminobenzamide HDAC inhibitors ameliorates the disease phenotype of a Friedreich ataxia mouse model. Neurobiol Dis. 2011;42(3):496–505. 10.1016/j.nbd.2011.02.016 21397024 PMC3107941

[28] Chutake YK, Lam CC, Costello WN, Anderson MP, Bidichandani SI. Histone Deacetylase Inhibitor Reverses Promoter Silencing in Friedreich Ataxia. Submitted (in review) 10.1093/nar/gkw107 PMC4914082 26896803

