# Supplementary material for: FXN Promoter Silencing in the Humanized Mouse Model of Friedreich Ataxia
Source: PLoS One. 2015 Sep 22;10(9):e0138437. doi: 10.1371/journal.pone.0138437 (PMC4579136; doi:10.1371/journal.pone.0138437)
Supplement: S1 Fig — (PDF) [file pone.0138437.s001.pdf]

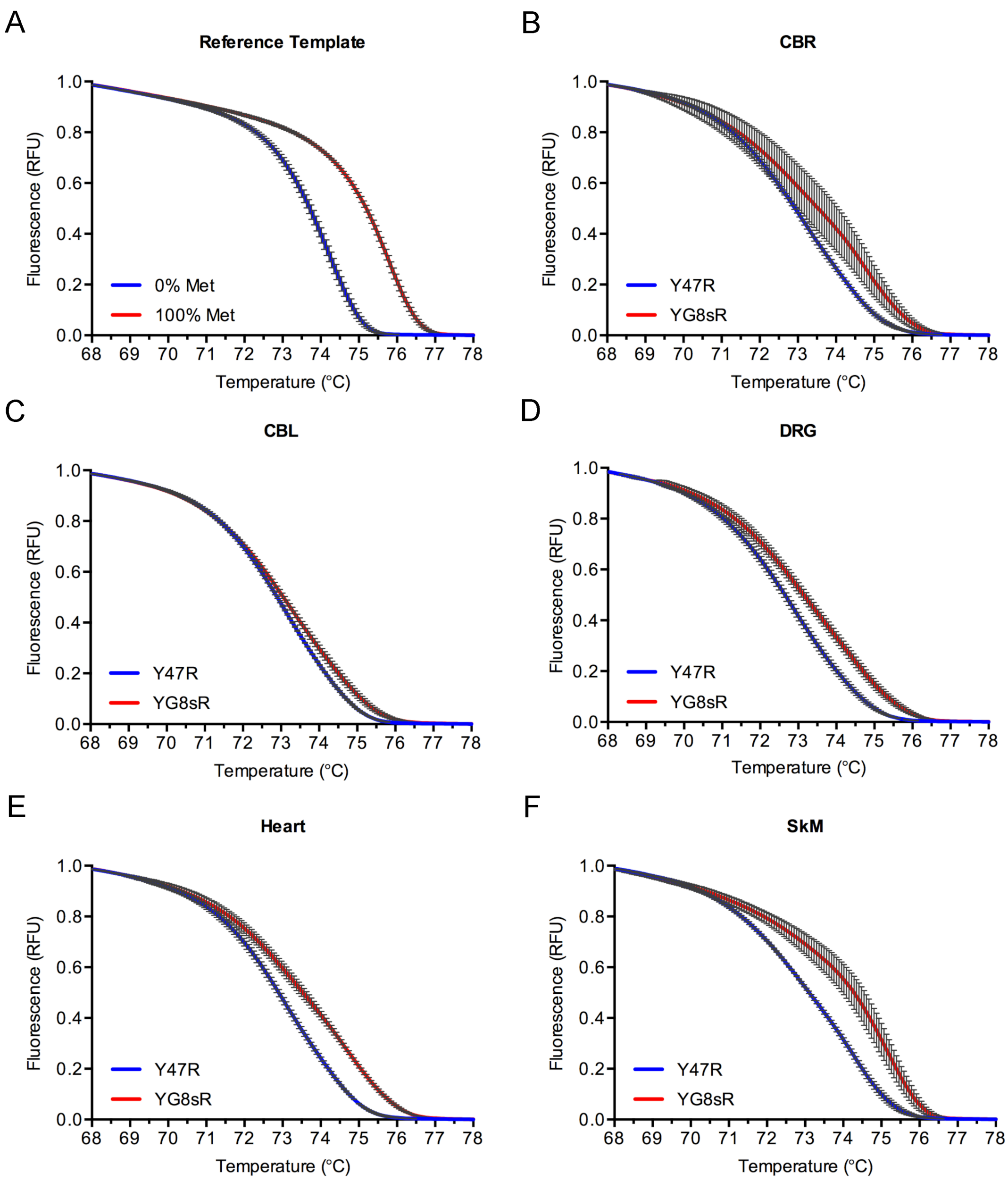

Supplementary Figure 1: Increased DNA methylation at the *FXN* locus in 1-month-old YG8sR mouse tissues. (A) Normalized melting curves in a high resolution melting (HRM) assay of two reference double-stranded templates simulating 100% (red curve) and 0% (blue curve) DNA methylation at three CpG sites upstream of the GAA-TR mutation (see Fig. 1A) showing a clear separation of the curves indicating that the HRM assay is able to detect methylation at the three CpG sites. **(B-F)** Normalized melting curves in a MS-HRM assay to detect CpG methylation in multiple tissues from 1-month-old YG8sR (red curves) and Y47R (blue curves) mice at the three CpG sites upstream of the GAA-TR mutation (see Fig. 1A) showing a clear separation of the curves indicating a relative increase in methylation at the three CpG sites in YG8sR tissues. For all HRM curves, X-axis = melting temperature, Y-axis = relative fluorescence, and error bars represent 95% confidence intervals at each of 15 points assayed in triplicate for fluorescence per °C change. CBR = cerebrum; CBL = cerebellum; DRG = dorsal root ganglia; SkM = skeletal muscle.
